# Periodontal maintenance: individual patient responses and discontinuations

**DOI:** 10.1186/s12903-022-02655-8

**Published:** 2022-12-15

**Authors:** Christian Graetz, Johannes C. Ehrenthal, Rebecca Kowalski, Miriam Cyris, Antje Geiken, Christoph E. Dörfer

**Affiliations:** 1 Clinic for Conservative Dentistry and Periodontology, University Hospital Schleswig-Holstein, Campus Kiel, Arnold-Heller-Str. 3, Haus B, 24105 Kiel, Germany; 2 Department of Psychology, University of Cologne, Cologne, Germany

**Keywords:** Adult attachment, Dental fear, Compliance, Periodontitis, Supportive periodontal therapy, Tooth loss

## Abstract

**Aim:**

There is a lack of data on long-term impact of different psychological variables on periodontitis. Aim of the current study was to investigate the impact of psychological factors in patients with chronic periodontitis (CP; according to the 1999 Classification of Periodontal Diseases) to explain adherence to or discontinuation of supportive periodontal therapy (SPT) in a university setting.

**Methods:**

A sample of n = 119 patients were examined in a questionnaire-based, cross-sectional survey. All patients had received active periodontal treatment (APT) and were reevaluated in a university setting (Kiel) before 2016 [T1: start SPT]. Patients who showed sufficient adherence to SPT of ≥ 2 years (maximum ± 6 months of deviation between SPT intervals, last visit and questionnaire at T2) were assigned to the adherence group (AG: n = 58), or, if they interrupted SPT or stopped treatment altogether, to the non-adherence group (NAG: n = 61). In addition to dental parameters, we assessed socio-demographic, treatment-related (critical attitudes/complaints), dental as well as psychological variables (especially psychological attachment, but also dental fear, patient participation style, personality functioning) and examined between-group differences as well as possible mediating factors of non-adherence to treatment continuation.

**Results:**

For both groups we found similar average observation time (NAG/AG: 15.9(8.9)/14.9(10.6)years). There were significant differences in age, critical attitudes, dental fear, and patient participation style between the groups. With the help of exploratory sequential mediation models, we found a significant indirect pathway of the impact of attachment anxiety on discontinuation of treatment mediated through dental fear and number of critical attitudes/complaints.

**Conclusion:**

Considering the limitations, dentists should be aware of personality-related risk-factors such as attachment anxiety as well as their interplay with levels of dental fear and critical attitudes which may influence adherence to SPT.

*Trial registration*: The clinical trial was retrospectively registered in the DRKS—German Clinical Trials Register (https://www.drks.de) with registration DRKS00030092 (26/08/2022).

## Background

More than 65% of the worldwide population are affects by periodontitis [1]. Untreated, the progression of periodontal destruction leads to functional and aesthetic constraints, discomfort, and tooth loss [2–4]. While early detection of periodontitis is crucial, yet difficult [5], implementation of adequate active periodontal therapy (APT) can stop disease- progression [6]. However, the chronic course of the disease also requires a supportive periodontal therapy (SPT) [7]. As with any chronic disease, compliance and continuation of treatment is essential, but at the same time especially challenging [8]. Inadequate information and motivational problems were identified as main patient-reported reasons for discontinuing SPT [9].

One approach to motives and behavior in medicine relates to psychological attachment. Attachment theory provides a bio-psycho-social model for predicting how individuals use interpersonal relationships to manage distress in situations of subjective threat, relating to stress management skills, health behavior, adherence to medical treatment and patient–physician interaction[10]. Psychological attachment describes a motivational-regulatory psychological structure which develops in the context of early repeated interactions with primary caregivers and stays relatively stable from infancy to high age [11]. In adults, different attachment styles can be distinguished. Securely attached individuals can rely on a deeply rooted trust that others will be available in times of distress, while insecure individuals are either uncomfortable relying on and getting close to others in times of need, usually called avoidant attachment, or view themselves as insufficient with respect to self-regulatory competence and try to obtain the attention and proximity of others, as described by anxious attachment. In periodontitis, attachment insecurity was related to for example healthcare-utilization [12] and it moderated the impact of the association between disease severity and dental fear [13]. High dental fear and low attendance was found in general dentistry for individuals with high levels of attachment insecurity in another, independent study [14].

To our knowledge, research on treatment discontinuation in specialized settings for periodontitis is rare [9] and the influence of psychological attachment pattern on treatment discontinuation during SPT is unclear [12]. Therefore, the aim of the questionnaire-based, cross-sectional survey was to investigate differences in attachment and other psychological factors in patients with periodontal disease in order to determine the reasons for adherence and non-adherence with long-term SPT in a university-based treatment-setting.

## Material and methods

### Sample

For recruitment we identified patients (n = 1217) with periodontitis who had been treated at our university hospital Kiel (Germany), and who (AG group) received APT (reevaluation at T1 with start of SPT before 31th of December 2016) and sufficient adherence for the following SPT of ≥ 2 years (max. ± 6 months of deviation between set SPT intervals, questionnaire at T2) or (NAG group) received APT phase and interrupted afterwards in SPT until 31th of December 2019. Potential subjects had to have clinical and radiographic documentation for sufficient diagnosis of a chronic periodontal (CP) disease according to the classification scheme valid at that time [15] and were willing to answer the questionnaires (period of the survey: 01/01/2019–12/31/2019). Therefore, we could control for the outcome of our active periodontal treatment and also for the time of SPT. Additional, in order to update the periodontal disease classification to the current scheme of 2018 [16], participants were re-classified according to periodontitis stages and grades based on the parameters [17]. However, this was only performed with regard to descriptive data as the necessary parameters for the documentation of the included patients was limited before 2018. We discuss this approach in more detail below.

From the n = 237 patients who met the inclusion criteria for the NAG group, n = 131 patients were not willing to participate in the study, n = 44 were not available by mail and/or displaced and n = 13 were deceased, resulting in a total sample of n = 61. Patients of the AG group were consecutive matched according to the number of teeth at T1 (± 1 tooth) and the time of SPT (± 10 years). Therefore, we could control for the outcome of our active periodontal treatment and also for the time of SPT.

Due to restrictions of the available database especially regarding the NAG group and the novelty of the research question, no sample size calculation for a specific test was performed a priori. Therefore, we included all available patients for the NAG condition and performed consecutive matching of eligible patients for the AG group on two key variables (n = 72). Of the 72 patients identified, 9 patients did not wish to participate in the study and 5 patients did not complete the questionnaire, leaving 58 patients in the AG group.

### Active and supportive periodontal therapy

Details of the current treatment concept are described elsewhere and analyzed, discussed, and compared to other university-based treatments [18]. Therefore, only a short overview is given here for:

*APT* included non-surgical, scaling and root planning (SRP) with, if indicated, additional access flap surgery. Further treatments, e.g. endodontic treatments, splinting of mobile teeth, adjunctive systemic antibiotics for seven days, regenerative or tunneling procedures and molar root resections were carried out in individual cases. Re-evaluation was planned after six months.

*SPT* followed an individualized interval of three to twelve months and included re-instruction/re-motivation of patients, individual oral hygiene, professional tooth cleaning with SRP of residual pockets and polishing by a dental auxiliary.

### Measures

#### Psychological attachment

Current attachment style (at T2) was assessed by self-report questionnaire following international standards [19], using a brief version of the German version of the Experiences in Close Relationships-Revised questionnaire (ECR-RD8) [20, 21]. The ECR-R is a widely used questionnaire with good psychometric properties that consists of 36 items rated on a scale from one to seven. It measures motives and behavioral tendencies concerning romantic relationships on two scales of attachment related anxiety and avoidance. The brief version ECR-RD8 used in this study assesses attachment anxiety and avoidance on four items, each with high reliability and validity [22]. Internal consistency was acceptable for anxiety (Cronbach’s α = 0.78) and high avoidance (Cronbach’s α = 0.90).

### Personality functioning

Levels of personality functioning as a dimensional approach to measuring personality disorders was conducted with the 12-item version of the Operationalized Psychodynamic Diagnosis-Structure Questionnaire (OPD-SQS, [23]). In this sample, internal consistency was good (Cronbach’s α = 0.87).

#### Participation preference

We assessed general preference to participate in clinical decision-making with the related six-item subscale of the modified German version of the Autonomy Preference Index (API-Dm; [24]). Internal consistency was in the lower range of acceptable (Cronbach’s α = 0.72).

#### Dental fear

For dental fear, the sum score of the Hierarchical Anxiety Questionnaire (HAQ; [25]) was used, which assesses subjective anxiety-related reactions to common patient experiences in dentistry. The HAQ comprises eleven items which are answered on a five-point scale (ranging from 1 = “relaxed” to 5 = “sick with fear”). Internal consistency was high (Cronbach’s α = 0.94).

#### Depression and anxiety symptoms

Depression was measured with the two-item screening instrument of the Patient Health Questionnaire (PHQ-2; [26]), anxiety with the related Generalized Anxiety Disorder (GAD-2, [27]). Internal consistency was high for both the PHQ-2 (Cronbach’s α = 0.84) and the GAD-2 (Cronbach’s α = 0.88).

#### Critical attitudes and complaints towards the treatment at the university clinic

To assess critical attitudes/complaints towards the university-based periodontal treatment, based on clinical experience and patient-related feedback, 18 items were presented to the participants on the question “What did you not like about the treatment in the hospital?”. Areas were as follows: Competence, thoroughness, character/personality of treatment staff, fluctuation of treatment staff, trust, treatment approach, treatment failure, treatment by dental students, appointing and waiting time, costs, parking lot and associated costs, paper work, treatment chairs, private reasons, travel/travel time. Circled areas were coded as one, non-circled areas as zero, and a sum score was calculated.

### Other variables

#### Health and oral hygiene survey

The health and oral hygiene survey (at T2) included general questions e.g. about age, smoking or drug intake. According to self-reported smoking history, patients were categorized. “Never smokers” had never smoked in their lives. Patients who had quit smoking continuously for at least 5 years back were looked upon as “former smokers”. All others were classified as current “smokers” [28].

#### Independent variables

Records of the medical history and the clinical charting were gender, age, severity of CP (light versus moderate-severe) according to the 1999 Classification of Periodontal Diseases [15], number of teeth at T1 and T2 and the number of teeth lost during observation time documented in a previously installed database. However, in many cases, the reasons for tooth removal were multiple, or could not be ascertained.

#### Data management and statistical analysis

All patients gave their informed consent for the analysis of their data documented during periodontal therapy. Data were sampled in a database (ParoDat, Department of Periodontology, Kiel, Germany), which was installed in 1982 and was set up on a database platform (FileMaker Inc., Santa Clara, USA) for continuous documentation of all patients. Descriptive analyses were conducted. In a first step, we calculated t-tests to assess possible differences in psychological and other variables between the groups and calculated effect sizes (Hedge’s g) to quantify the size of those differences. Wherever, according to Levene’s test indicated a violation of equality of variances, we used a statistic that does not assume homogeneity of variances. For comparing gender, smoking status, and relationship status, we used χ2-tests. In a second step, we calculated zero-order correlations between variables that showed the largest differences between the groups and other variables. In a third step, we explored a model of psychological variables possibly associated with treatment discontinuation. For that, we used bootstrapping-based (m = 5000) sequential moderation. All analyses were conducted with IBM SPSS 27 (SPSS, Chicago, IL, USA) and the PROCESS macro version 3.4.

## Results

### Sample and tooth loss

At start of SPT at T1, the included 61 patients in the NAG group (male/female: 24/37) had a mean(SD) age of 48.2(11.3) (range: 26–72) years, and a permanent dentition with a mean of 25.1(3.9) (range: 15–31) teeth. The 58 patients of the AG group (male/ female: 32/26) were at T1 55.7(11.6) (range: 27–75) years old, and showed a permanent dentition with a mean of 25.5(3.6) (range: 14–31) teeth. In NAG 9 (14.8%) patients were classified with light and 52 (55.2%) with moderate-severe CP (AG: 13 (22.4%) of light/45 (77.6%) of moderate-severe CP). After updating the classification of periodontal disease into the current scheme of 2018 in stages and grades [17] we found 9 patients (14.7%) in NAG were classified with stage II (grade A/B/C: 2/3/4), 32 (52.5%) with stage III (grade A/B/C: 0/12/20) and 20 (32.8%) with stage IV (grade A/B/C: 0/2/18) (AG: 11 patients (19.0%) stage II (grade A/B/C: 2/6/3), 35 (60.3%) with stage III (grade A/B/C: 0/22/13) and 12 (20.7%) with stage IV (grade A/B/C: 0/5/7)). Equal distribution was found for localized versus generalized diseases in both classification systems. Details are summarized in Table 1.

**Table 1:** Characteristics of patients at different time points in both groups of non-adherence with SPT (NAG) versus adherence with SPT (AG) in a university setting

	NAG	AG
N of patients (male/female)	24/37	32/26
Age at T1 (mean (SD) (range) in years	48.2(11.3) (range: 26–72)	55.7(11.6) (range: 27–75)
N of patients with light/ moderate-severe CP	9/52	13/45
N of patients with stage II (grade A/B/C)	9 (2/3/4)	11 (2/6/3)
N of patients with stage III (grade A/B/C)	32 (0/12/20)	35 (0/22/13)
N of patients with stage IV (grade A/B/C)	20 (0/2/18)	12 (0/5/7)
N of patients with localized/generalized periodontal disease	45/16	34/24
Time of SPT (mean (SD) (range) in years	15.8(8.2) (range: 1.5–24.9)	14.9(10.6) (range: 2.3–36.9)
N of teeth at T1 (mean (SD) (range)	25.1(3.9) (range: 15–31)	25.5(3.6) (range: 14–31)
N of teeth/last entry in medical record in SPT (mean (SD) (range)	22(6.4) (range: 2–30)	23.2 ± 5.2 (range: 7–31)
N of teeth at T2 (mean (SD) (range)	21(7.1) (range: 2–34) (self-reported)	22.3(6.3) (range: 8–32)
N of patients with tooth loss 0/1–3/4–6/≥ 7 during observation time	19/ 22/ 13/ 7 (self-reported)	17/ 29/ 5/ 7
N of tooth loss at T2 (mean(SD) (range)	3.1(4.3) (range: 0–24) (calculated according self-reported T2 and data of T1)	2.3(2.9) (range: 0–12) (calculated according documentation in clinical charts)
N of smoker/former smoker/never smoker (T1)	18/18/25	13/21/24
N of smoker/former smoker/never smoker (T2)	18/18/25	12/22/24
N of cigarettes (mean(SD) (range) per day for active smoker at T2	13.4 ± 9.8 (range: 0–40)	12.9 ± 4.5 (range: 4–20)
N of stops smoking (mean(SD) (range) for active smoker at T2	3.3(2.6) (range: 1–10)	2.4(3.4) (range: 1–10)
N of patients with DM at T1 (mean(SD) (range)	2	3
HbA1c (glycated haemoglobin) at T1 (mean(SD) (range)	7.7(1.5) (range: 6.6–8.7)	7.0(1.1) (range: 6.0–8.1)/
N of patients with DM at T2 (mean(SD) (range)	4	4
HbA1c (glycated haemoglobin) at T2 (mean(SD) (range)	6,7(0.4) (range: 6.1–7.1)	6.5(0.5) (range: 6.0–7.1))

CP: chronic periodontitis; SPT: supportive periodontal therapy; NAG: patients´ group discontinued the SPT at the department of periodontology, Kiel; AG: patients’ group with regular SPT at the department of periodontology, Kiel; T1: start of SPT at the department of periodontology, Kiel; T2: questionnaire and in AG last visit in SPT

Two NAG patients reported a diabetes mellitus (DM) disease at T1 (AG: 3) and at T2 in total four DM patients reported a DM (AG: n = 4). At T1 in the NAG group 25 (41.0%) patients reported non‐smoking, 18 (29.5%) former smoking and 18 (29.5%) active smoking (in AG n (%) of non/former /active smokers: 24 (41.4%)/ 21 (36.2%)/ 13 (22.4%)). During observation time T1-T2 no NAG patients changed smoking habit, whereat in AG two patients stop smoking and one former smoker starts again to smoke (in AG n (%) of non/former /active smokers at T2: 24 (41.4%)/ 22 (37.9%)/ 12 (20.7%)).

As determined by inclusion, in AG all patients showed sufficient adherence with SPT of 14.9(10.6) [range: 2.3–36.9] years (T1-T2). In the NAG group, as a prerequisite all patients discontinued their SPT follow-up in our department after 15.85 (8.92) (range: 1.5–24.9) years and declared by themselves that no further systematic maintenance program was visited afterwards. Nineteen (31.1%) of the NAG patients reported no tooth lost during observation time (AG: n = 17 (29.3%)). The calculation according to these self-reported number of teeth at T2 resulted in NAG in an average of 3.1(4.3) (range: 0–24) teeth lost during observation time (in AG calculation was performed with clinical data at T2 and T1 from our charts: 2.3(2.9) (range: 0–12) teeth lost).

### Differences between the groups

The NAG group was characterized by a significantly lower age at the start of the SPT (medium effect), significantly higher dental fear (small to medium effect), significantly higher wish for participation in treatment decision (medium effect), a trend towards more general anxiety (small effect), and higher number of critical attitudes/complaints towards the university-based treatment (large effect). There were no significant differences regarding gender (χ^2^ = 2.99, p = 0.10), relationship status (χ^2^ = 5.85, p = 0.21), or smoking status (χ^2^ = 2.82, p = 0.24) between the groups. For more information, see Table 2.

**Table 2:** Differences in variables between patients of non-adherence with SPT (NAG) versus adherence with SPT (AG) in a university setting

Variable	Group	N	Mean (SD)	T	Effect size
Age at T1	AG	58	55.67 (11.60)	3.54**	0.65
	NAG	61	48.23 (11.36)		
Years in SPT	AG	58	14.98 (10.61)	− 0.49	0.09
	NAG	61	15.85 (8.92)		
Number of teeth at T1	AG	58	25.48 (3.55)	0.62	0.11
	NAG	61	25.05 (4.00)		
Loss of teeth during observation time (T2–T1)	AG	58	2.29 (2.92)	− 1.20	0.22
	NAG	61	3.10 (4.32)		
Number of critical attitudes/complaints towards the treatment	AG	58	0.09 (0.28)	− 6.16***	1.12
	NAG	61	1.00 (1.10)		
Dental fear (HAQ)	AG	56	17.81 (7.44)	2.31*	0.43
	NAG	58	21.29 (8.58)		
Depression (PHQ-2)	AG	51	0.39 (1.10)	− 1.50	0.29
	NAG	54	0.72 (1.16)		
Anxiety (GAD-2)	AG	50	0.32 (1.02)	− 1.95´	0.38
	NAG	54	0.74 (1.19)		
Participation (API)	AG	52	15.45 (4.31)	− 3.24**	0.62
	NAG	58	18.28 (4.78)		
Personality functioning (OPD-SQS)	AG	50	8.94 (6.76)	− 0.87	0.17
	NAG	57	10.19 (7.86)		
Psychological attachment anxiety (ECR-RD8)	AG	41	1.71 (1.23)	-1.17	0.25
	NAG	48	2.04 (1.36)		
Psychological attachment avoidance (ECR-RD8)	AG	42	3.46 (2.61)	0.43	0.09
	NAG	48	3.25 (2.06)		

´p > 0.1; *p > 0.05; **p > 0.01; ***p > 0.001

SPT: supportive periodontal therapy; NAG: patients´ group discontinued the SPT at the department of periodontology, Kiel; AG: patients’ group with regular SPT at the department of periodontology, Kiel; T1: start of SPT at the department of periodontology, Kiel; T2: questionnaire and in AG last visit in SPT; API: Autonomy Preference Index; GAD-2: anxiety items of the Patient Health Questionnaire; PHQ-2: depression items of the Patient Health Questionnaire; HAQ: Hierarchical Anxiety Questionnaire; ECR-RD8: Short version of the Experiences in Close Relationships – Revised questionnaire; OPD-SQS: Operationalized Psychodynamic Diagnosis—Structure Questionnaire

### Associations between variables and sequential mediation

We correlated critical attitudes as the variable with the most impact on the difference between the two groups, with all variables already displayed in Table 2. Number of critical attitudes and complaints was significantly associated with age at first admission (r = − 0.31, p = 0.001), HAQ dental fear (r = 0.45, p < 0.001), PHQ-2 depression (r = 0.22, p = 0.028), GAD-2 anxiety (r = 0.23, p = 0.018), OPD-SQS personality functioning (r = 0.25, p = 0.009) and ECR-RD8 attachment anxiety (r = 0.25, p = 0.017).

In a next step, we tested sequential mediation models to statistically predict group status (discontinued vs. continued SPT). As the first predictor we used ECR-RD8 psychological attachment-related anxiety as relatively stable personality-oriented variable and as the last predictor critical attitudes/ complaints. Due to correlations with all other variables of interest and prior findings [13], we included HAQ dental fear as a more state-related factor. We subsequently exploratively added other variables that were significantly associated with both attachment anxiety and critical attitudes as potential mediators and covariates. The final model consisted of attachment anxiety, dental fear, and critical attitudes as predictors and group status as outcome, while controlling for ECR-RD8 psychological attachment avoidance, age at first admission to the treatment clinic, and the API participation subscale.^1^

**Fig. 1:**
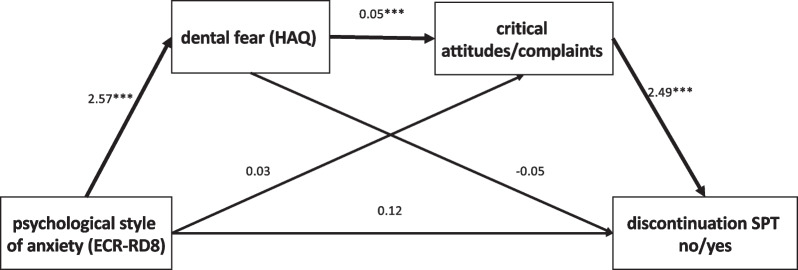
Sequential mediation of psychological factors on the association between psychological attachment anxiety and SPT discontinuation. ´p > 0.1; *p > 0.05; **p > 0.01; ***p > 0.001. *SPT* supportive periodontal therapy, *HAQ* Hierarchical Anxiety Questionnaire, *ECR-RD8* Experiences in Close Relationships-Revised questionnaire

There was a significant indirect effect of ECR-RD8 attachment anxiety on discontinuation of specialized university treatment mediated via HAQ dental fear and number of critical attitudes and complaints about areas of the treatment format and institution. There was no significant direct effect of ECR-RD8 attachment anxiety on treatment discontinuation (effect = 0.12, Z = 0.44, LLCI = − 0.44, ULCI = 0.68). Only the path of ECR-RD8 on treatment discontinuation via HAQ and critical attitudes/complaints yielded a significant indirect effect (effect = 0.35, LLCI_bootstrapped_ = 0.10, ULCI_bootstrapped_ = 1.19). For the exact results, please see Table 3 and Fig. 1.

**Table 3:** Sequential mediation of psychological factors on the association between psychological attachment style of anxiety and SPT discontinuation

Path/variables	Coefficient	t/Z	LLCI	ULCI
Psychological style of anxiety (ECR-RD8) → dental fear (HAQ)	2.57	4.12	1.33	3.80
Psychological style of anxiety (ECR-RD8) → critical attitudes/complaints	0.03	0.43	− 0.13	0.19
Psychological style of anxiety (ECR-RD8) → discontinuation of SPT	0.12	0.44	− 0.44	0.68
Dental fear (HAQ) → critical attitudes/complaints	0.05	4.20	0.03	0.08
Dental fear (HAQ) → discontinuation of SPT	− 0.05	− 1.14	− 0.15	0.04
Critical attitudes/complaints → discontinuation of SPT	2.49	3.69	1.17	3.82

LLCI: lower level of confidence interval; ULCU: upper level of confidence interval; SPT: supportive periodontal therapy; HAQ: Hierarchical Anxiety Questionnaire; ECR-RD8: Short version of the Experiences in Close Relationships-Revised questionnaire. All paths controlled for ECR-RD8 avoidance, age at initial admission to specialized treatment, and API participation subscale. Paths predicting treatment discontinuation relied on Z-values due to the dichotomous nature of the variable, all other paths on t-values

## Discussion

The aim of our study was to investigate the impact of psychological and dental variables on discontinuation of SPT in patients with periodontitis, given the relevance of ongoing periodontal maintenance after successful periodontal treatment [6] for favorable long-term outcome. In previous studies we could show that different university-based treatment regimens resulted in lower tooth-loss during SPT [18].

Adherence to SPT can be influenced by different psychological factors [12] which could be grouped into (a) directly affecting, b) indirectly affecting state-oriented, and (c) indirectly affecting trait-oriented variables. Directly affecting variables are factors that are closely related to treatment acceptance and organization and consciously accessible for patients, which are mostly summarized in the critical attitudes against treatment in our university setting. Indirect state-orientated factors comprise variables that have the potential to affect treatment compliance, but are less directly attributed to characteristics of the treatment setting. Anxieties regarding treatment needs for example to be managed [29], otherwise patients will not continue attending treatment appointments [30]. The indirect trait-oriented factors are relatively stable psychological variables that serve as general, distal risk-factors in health-behavior, for example psychological attachment insecurity. In our data we found a significant indirect path of attachment anxiety on discontinuation of treatment mediated through dental fear and number of critical attitudes/complaints (Fig. 1). Attachment anxiety is known to influence general anxiety [31, 32] as well as dental-related fear [14]. While anxiety my not generally be related to non-compliance [33], an accompanying critical attitude can either be prospectively relevant for discontinuation, or retrospectively used to reduce cognitive dissonance for the decision to terminate SPT. Note that in our study we matched time of SPT of both groups, which allows us to control for example for effects of early drop-out (i.e., “non-starters”). While we therefore cannot infer this from our study, attachment insecurity, though a normal facet of development [34], may narrow health-related regulatory strategies, serve as a risk factor concerning stress regulation, and is directly linked to chronic somatic health conditions [35].

While attachment avoidance may lead to a late start of the APT [12] individuals with attachment anxieties tend to follow all necessary steps at the beginning of the therapy, but have a high risk of discontinuation. Whereas attachment avoidance can be counterparted by individualized motivation programs for adherence, smoking cessation or promotion of healthy lifestyles [36] patients with attachment anxiety may be kept in therapy by continuous information and motivation during the periodontal therapy to [1] increase patient adherence [9] by avoiding their overdependence [37] and to [2] reduce misunderstanding or extreme treatment expectations with the consequence of overtreatments [38]. Shah et al. [39] measured a correlation between the patient's oral health awareness (OHA) and number of missed appointments in SPT. As OHA should be modified in early periodontal treatment [6], this will help to improve understanding the necessity of supporting patients with attachment anxiety, dental fear or critical attitude to make adherence to SPT more predictable [39].

In our previous investigation with 310 compliant patients, we found that women with higher attachment anxiety had more visits during SPT [12]. In the current study we couldn’t detect significant differences between men and women, which may be due to the size of the study, as it differs to other findings showing small gender effects [40, 41].

Although overall scores for attachment avoidance, modelling the entrance into the therapy, were higher than for anxiety, modelling the persistence in the therapy independent of the respective group (Table 2) in long-lasting therapies such as the therapy of periodontitis the experiences of stigmatization, rejection and loneliness [42, 43], or even impaired use of positive interactional cues, such as smiling [44] are essential for adherence. Maintaining adherence over long-term SPT is and remains a challenge, not only in private practice [45, 46] but, as the current results show, also in a special cohort of patients in a university setting. The results of our study may give hints on how the adherence of patients may be improved even under less optimal psychological conditions.

Although our study is based on a well-characterized sample of individuals, there are some limitations.^2^ A sample size of 119 patients seems tolerable for a study like this. However, especially the subgroup-analyses need replication in larger studies to guarantee adequate statistical power. The limitation of a selection bias is obvious, but as already pointed out makes it more likely not to find any differences. Seeing significant differences despite this weakening factor may support the importance of attachment anxiety on the adherence to SPT. A last point to mention, the initial diagnosis of periodontitis in the current investigation was based on the baseline examination, anamnesis, radiographs as well as any additional information documented in our database according to the 1999 classification [15]. The correction of diagnosis according to the 2018 classification [16] was only possible to a limited extent for the included patients, who were already admitted several years before the current investigation. Some of the essential criteria for the assessment of staging and grading [17] were neither recorded nor documented in our database in previous time (e.g., adjustment of the HbA1c) and can also not be queried subsequently (specifically in the non-adherent group). Therefore, and in line with other previously published results of long-term data regarding adherence in SPT [47], we re-classified the patients only for descriptive purpose but not for further analysis. We found the new classifications came with a significant class imbalance in our specific cohort with less severe stages (I-II) and grades (A) being underrepresented. This trend has already been observed in several of our patient cohorts analyzed in other studies [48, 49]. Thus, a relatively large number of patients with severe periodontal disease were treated in our university setting. This may not be the case in other populations and settings. On the other side, it should be noted that while the 1999 classification was able to reflect patients’ characteristics, classes showed only limited differences with regard to disease risk and complexity factors, which will influence approaches to therapy and disease outcomes [48]. However, from a point of reliability of classification, we are as confident as possible that our university setting and long-term treatment of most of the patients provides a solid base for high-quality assessment. In general, large prospective study designs with multiple measurement points of dental as well as psychosocial variables are needed in dentistry.

## Conclusion

In conclusion and within the limitations of this study, we were to describe a model, how attachment anxiety is related to discontinuation during SPT. This is of special relevance, as dentists should be aware of attachment anxiety as an indirect factor which could influence via dental fear and critical attitudes the adherence during maintenance.

## Data Availability

The datasets used and/or analysed during the current study are available from the corresponding author on reasonable request.

## Abbreviations

AG: Adherence group
API: Autonomy Preference Index
APT: Active periodontal therapy
CP: Chronic periodontitis
DM: Diabetes mellitus
ECR-RD8: Short version of Experiences in Close Relationships-Revised
GAD-2: Anxiety items of the Patient Health Questionnaire
HbA1c: Glycated haemoglobin
HAQ: Hierarchical anxiety questionnaire
LLCI: Lower level of confidence interval
NAG: Non-adherence group
OHA: Oral health awareness
OPD-SQS12: Operationalized Psychodynamic Diagnosis-Structure Questionnaire
PHQ-2: Depression items of the Patient Health Questionnaire
SPT: Supportive periodontal therapy
T1: Start of supportive periodontal therapy at the department of periodontology
T2: Questionnaire and in adherence group last visit in supportive periodontal therapy
ULCU: Upper level of confidence interval

## Footnotes

1 ECR-RD8 avoidance was included as a covariate from a conceptual point of view, to rule out that merely attachment insecurity, but not specific attachment anxiety drove the effect; age at first admission and API participation were included as they added to the model on a trend level (p < 0.1).

2 Taking up a reviewer’s comment, while we do not think this was a major issue in our study, we cannot rule out general survey response fatigue effects. This needs further research in all areas of medicine.
